# The Impact of Peer Support Through Mobile Health Applications on Breastfeeding Outcomes: A Systematic Review and Meta‐Analysis

**DOI:** 10.1111/mcn.70249

**Published:** 2026-09-21

**Authors:** Yim Ching Yau, Mary Fewtrell, Martin Ming Him Wong, Ping Him Lam, Jinyue Yu, Kerrie Stevenson

**Affiliations:** ^1^ Institute of Epidemiology and Health Care University College London London United Kingdom; ^2^ Great Ormond Street Institute of Child Health University College London London United Kingdom; ^3^ Department of Computing Room PQ806, Mong Man Wai Building, The Hong Kong Polytechnic University Kowloon Hong Kong SAR China; ^4^ Faculty of Population Health Sciences University College London London United Kingdom

**Keywords:** breastfeeding; peer support, exclusive breastfeeding, maternal health, meta‐analysis, mHealth, mobile applications, systematic review

## Abstract

Fewer than 40% of infants are exclusively breastfed for the first 6 months despite global recommendations, hindered by socioeconomic, cultural, and systemic barriers. Mobile health (mHealth) technologies provide scalable breastfeeding support, but the effectiveness of digital peer support across settings remains underexplored. This systematic review and meta‐analysis searched PubMed, Embase, CINAHL, Web of Science, and the Cochrane Library for randomised controlled trials (RCTs) published between 1 January 2010 and 1 May 2025 (PROSPERO reference number: CRD420251164765). Studies involved pregnant or postpartum women (up to 12 months) receiving mHealth peer support compared with standard care or non‐peer digital interventions. Primary outcomes were exclusive and any breastfeeding rates; secondary outcomes included self‐efficacy, duration, frequency, initiation, attitudes, and satisfaction. Data were narratively synthesised, and pooled estimates calculated via random‐effects meta‐analysis. From 3102 identified records, seven RCTs involving 3591 participants were included, of which five studies were eligible for quantitative meta‐analysis. Interventions delivered peer support through mobile apps, phone calls, group chats, mentoring, and real‐time messaging. The meta‐analysis found no significant effect on any breastfeeding at the primary time point (RR = 1.07, 95% CI 0.95–1.21; *I* ^2^ = 55.0%), but it did significantly improve exclusive breastfeeding at 4–6 months postpartum (RR = 1.13, 95% CI 1.01–1.26; *I* ^2^ = 0%). Leave‐one‐out sensitivity analyses supported the robustness of both estimates, although the lack of heterogeneity for exclusive breastfeeding should be interpreted cautiously because of the small number of studies. Narrative findings also showed localised gains in overall breastfeeding and greater maternal confidence, satisfaction, and perceived support, especially with interactive and culturally relatable peer support. mHealth peer support shows promise for improving exclusive breastfeeding, especially at 4–6 months postpartum, but evidence for any breastfeeding is less consistent and mostly limited to HICs/UMICs. Future studies should focus on LMICs, hybrid models, long‐term follow‐up, process evaluation, and cost‐effectiveness.

## Summary

- Peer support delivered through mobile health (mHealth) applications significantly improves exclusive breastfeeding (EBF) at 4–6 months postpartum. However, its overall impact on any breastfeeding remains directionally favourable but inconclusive; while individual trials reported significant gains, the pooled global effect was not statistically significant. High maternal satisfaction and recommendation rates (up to 100%) reported.
- Synthesised evidence includes seven randomised controlled trials (RCTs) with 3591 participants, primarily conducted in high‐ and upper‐middle–income countries (HICs/UMICs), highlighting a total absence of data from low‐income settings (LMICs).
- Heterogeneity was absent for exclusive breastfeeding (*I* ^2^ = 0%), though interpreted cautiously due to the small number of studies. In contrast, moderate heterogeneity was observed for any breastfeeding (*I* ^2^ = 55.0%).
- Future research should target LMICs, use hybrid (digital and real‐time video) formats, conduct process and cost‐effectiveness evaluations, and monitor long‐term breastfeeding outcomes.

## 1 Introduction

Breastfeeding is a cornerstone of early‐life nutrition and a globally recognised public health priority that confers substantial benefits for both infants and mothers (World Health Organisation 2024). It reduces infant morbidity and mortality, promotes cognitive development, and offers protection against infectious diseases such as diarrhoea and pneumonia, while also lowering maternal risks of breast and ovarian cancers and supporting healthy birth spacing (Rollins et al. 2016; Victora et al. 2016; World Health Organisation 2024). The World Health Organisation (WHO) and United Nations Children's Fund (UNICEF) recommend exclusive breastfeeding (EBF) for the first 6 months of life, followed by continued breastfeeding alongside complementary foods for up to 2 years or beyond (UNICEF 2023; World Health Organisation 2017 & 2024). Despite its established benefits, global EBF rates remain suboptimal, with fewer than 40% of infants under 6 months being exclusively breastfed and well below the 2030 global target of 70% (Gertosio et al. 2016). Multifactorial barriers, including inadequate skilled support, early return to work, sociocultural norms, misinformation, and unsupportive healthcare environments, continue to hinder optimal breastfeeding practices (Dowling et al. 2012; Kavle et al. 2017; McFadden et al. 2017).

Peer support has emerged as a vital complementary strategy to professional care in addressing these challenges and promoting breastfeeding continuation. It is assistance, empathy, and encouragement offered by women with personal breastfeeding experience, who provide emotional, informational, and appraisal support to new or expectant mothers (Dennis 2003; Evans et al. 2025; Jolly et al. 2012). Qualitative evidence indicates that breastfeeding peer support enhances women's confidence and reduces isolation by providing empathetic, experience‐based assistance (Chang et al. 2022). McFadden et al. (2017) analysed 100 trials (73 contributing data from 74,656 mother‐infant pairs across 29 countries), but the evidence specifically relevant to peer or lay support came from a smaller subset of trials. In that review, lay support interventions were associated with reduced cessation of EBF in some analyses, whereas the meta‐analysis for any breastfeeding at 4–6 weeks was not statistically significant. Shakya et al. (2017) highlight that community‐based in‐person peer support effectively boosts EBF duration for mothers, especially among infants aged 3–6 months in low‐ and middle‐income countries. Despite the potential benefits, traditional peer support programmes often encounter limitations related to sustainability, resource intensity, and geographic accessibility, particularly in low‐resource and rural settings (Brown and Tennant‐Eyles 2021; Chepkirui et al. 2020; Evans et al. 2025; Morse and Brown 2021; Read and McGrath 2018; Trickey 2018). These constraints underscore the need for innovative, scalable strategies that can deliver consistent and contextually relevant breastfeeding support worldwide.

The proliferation of mobile health (mHealth) technologies presents an opportunity to expand the reach and impact of peer support interventions. mHealth applications enable the delivery of tailored, interactive, and real‐time breastfeeding support through mobile platforms, integrating features such as text messaging, group forums, and peer mentoring networks (Lee et al. 2015; McKay et al. 2018; Sondaal et al. 2016). By fostering community interaction and social connectedness, digital peer support interventions can enhance maternal motivation, reinforce breastfeeding knowledge, and promote sustained engagement (Chen et al. 2018; Pratiwi et al. 2023). Recent studies suggest that some mHealth‐based peer support may improve breastfeeding self‐efficacy and exclusivity rates, but the evidence remains inconsistent due to heterogeneity in design, outcome measures, and contextual implementation (Diniz et al. 2019; Leal et al. 2023; Yau et al. 2026; Ziebart et al. 2024).

Furthermore, the effectiveness of digital peer networks is influenced by factors such as digital literacy, access inequities, content quality, and cultural alignment. Limited smartphone ownership, poor internet connectivity, and inconsistent moderation practices can restrict participation and lead to variable intervention fidelity, particularly in low‐ and middle‐income countries (Knop et al. 2024; Mieso et al. 2022; Qian et al. 2021). Despite an expanding evidence base on mHealth and breastfeeding, systematic synthesis focusing specifically on peer support mechanisms integrated within mobile applications remains scarce. Previous reviews have primarily examined digital education or professional‐led counselling, with limited exploration of peer‐driven engagement and behavioural change processes.

To address this knowledge gap, the present systematic review and meta‐analysis aims to evaluate the effectiveness of peer support delivered through mobile health applications in promoting breastfeeding worldwide. Specifically, it seeks to: (1) identify and classify the types of mHealth‐based peer support interventions implemented, (2) assess their impact on key breastfeeding outcomes such as initiation, exclusivity, and duration, and (3) examine the facilitators and barriers influencing their effectiveness across diverse sociocultural and economic contexts. Table 1 presents key definitions applied throughout this paper.

**Table 1:** Definitions of key terminologies.

Terminologies	Definitions
mHealth	Use of mobile devices and wireless technology to support medical and public health practices (World Health Organisation 2011).
Peer support in breastfeeding	Assistance, empathy, and encouragement offered by women with personal breastfeeding experience, who provide emotional, informational, and appraisal support to new or expectant mothers (Evans et al. 2025; Jolly et al. 2012).
Mobile application‐based interventions	Interventions or programmes delivered through mobile phone applications to support medical and public health practices (World Health Organisation 2011), including app‐based, messaging‐based, and fully telephone‐based peer support approaches, to support breastfeeding and related maternal health practices.
Exclusive breastfeeding (EBF)	Feeding only breast milk (no food or drink) (World Health Organisation 2024). (The study will also adopt the definitions of EBF as used in each included study to ensure comparability and inclusivity of available evidence.)
Breastfeeding Self‐efficacy	A mother's confidence in her ability to breastfeed her new infant (Dennis 1999).
Effectiveness	The extent to which an intervention achieves its intended outcomes in real‐world settings (Rychetnik et al. 2002).
Upper‐middle or High‐Income Countries (UMICs and HICs)	Countries with a GNI per capita greater or between $4,495 and $13,935 (The World Bank 2024).
Low‐ and Middle‐Income Countries (LMICs)	Countries with a GNI per capita lower or between $1,136 and $4,495 (The World Bank 2024).
Telelactation	Services that connect breastfeeding mothers to remotely located healthcare professionals using any real‐time audio‐visual technology (Kapinos et al. 2019).

## 2 Methods

This systematic review and meta‐analysis was conducted in accordance with the Preferred Reporting Items for Systematic Reviews and Meta‐Analyses (Preferred Reporting Items for Systematic Reviews and Meta‐Analyses PRISMA 2025) guidelines. The search strategy was guided by the Population, Intervention, Comparator, Outcome (PICO) framework (Supporting Information S1: Materials 1 and 2) and structured around three principal concepts: *breastfeeding*, *peer support delivered through mHealth/mobile applications*, and *study design*. Search terms incorporated relevant Medical Subject Headings (MeSH), truncations, synonyms, and variant spellings, which were combined using Boolean operators to enhance sensitivity and specificity.

A comprehensive literature search was undertaken across PubMed, Embase, CINAHL, Web of Science, and the Cochrane Library for studies published between 1 January 2010 and 1 May 2025. This period was chosen to capture research conducted during the widespread adoption of smartphones and mHealth technologies, while allowing sufficient time for studies to be published and indexed. The search was supplemented by manual screening of reference lists from key reviews and commentaries. All references were managed using Rayyan version 1.4.3 (2025), and duplicates were removed prior to screening.

### 2.1 Eligibility Criteria

Eligibility criteria were defined a priori in consultation with study authors, domain experts, and existing evidence. Studies were included if they met the following criteria:

- Population: Pregnant women or postpartum mothers (up to 12 months following delivery), regardless of parity or infant health status, including preterm infants.
- Intervention: mHealth or mobile application–based interventions that incorporated peer support components (e.g., group chats, peer mentoring, peer counselling, or community forums) aimed at promoting breastfeeding. Peer supporters were lay individuals (non‐professionals) with prior breastfeeding experience; formal training of peer supporters was not required as an inclusion criterion.
- Comparator: Standard care, other digital interventions without peer support, or no intervention.
- Outcomes: At least one breastfeeding‐related outcome (initiation, exclusivity, or duration of breastfeeding; maternal self‐efficacy, attitudes or knowledge).
- Study design: Randomised Controlled Trials (RCTs) published in English.

Studies were excluded if they:

- Targeted only healthcare professionals or family members without direct focus on mothers.
- Relied exclusively on face‐to‐face or hybrid peer support without mobile components.
- Used non‐randomised, observational, or qualitative designs.
- Full text unavailable.
- Did not report relevant breastfeeding or behavioural outcomes.

### 2.2 Study Selection and Data Extraction

Titles and abstracts were screened in duplicate by two independent reviewers (CY and MW/LP/KS). Full texts were retrieved and assessed for eligibility using a structured checklist aligned with inclusion and exclusion criteria. Discrepancies between reviewers were resolved via discussion or adjudication by a third reviewer. Reasons for exclusion at the full‐text stage were systematically documented.

**Figure 1:**
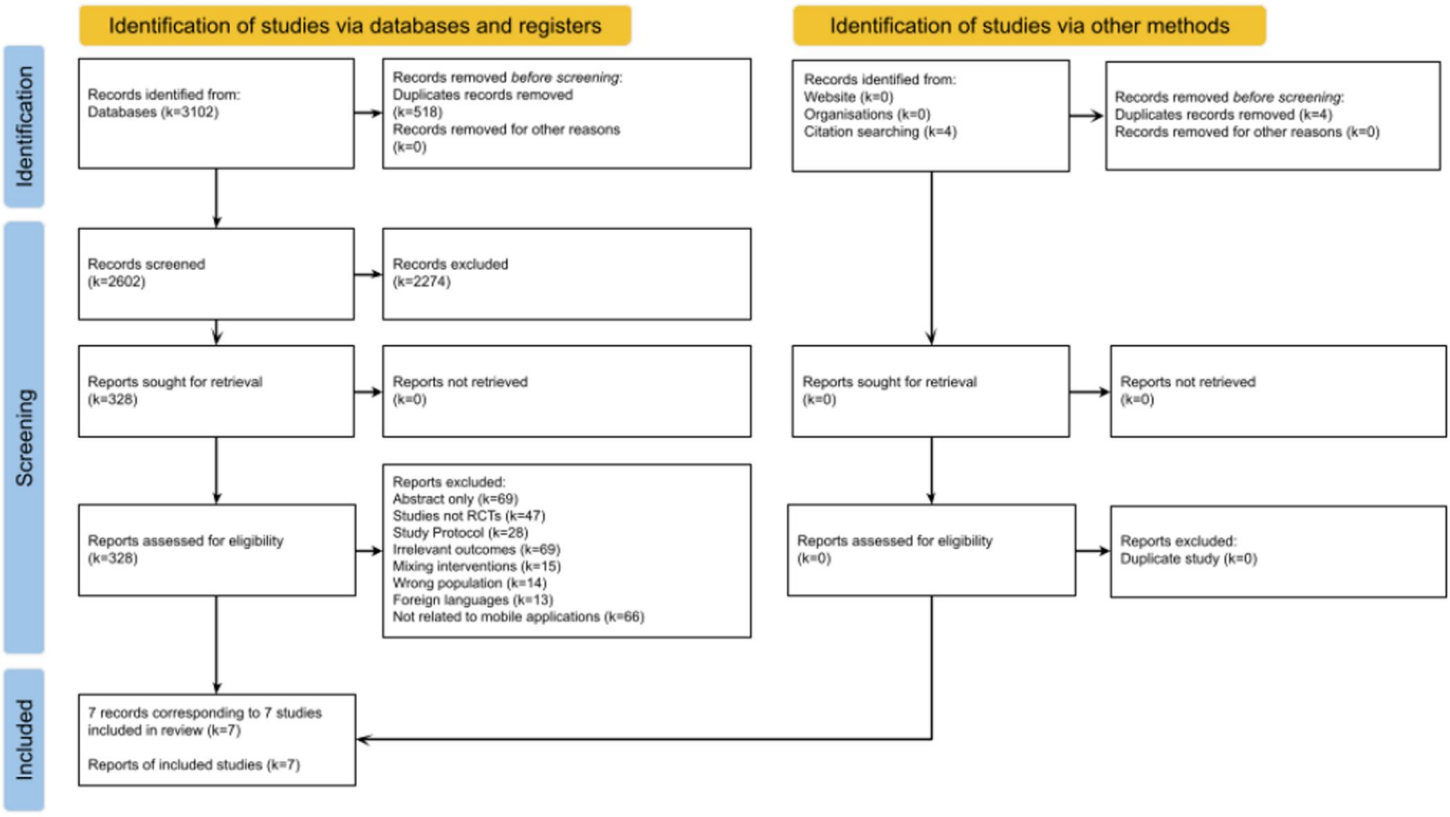
Preferred reporting items for systematic reviews and meta‐analysis (PRISMA) flow diagram (k = number of studies). Of the seven studies meeting the inclusion criteria, two studies (Meglio et al. 2010; Reeder et al. 2014) were retained in the narrative synthesis but excluded from the quantitative meta‐analysis. Meglio et al. (2010) was excluded because the study had substantial and differential attrition, and the intervention delivery itself was unstable, which together raised some concerns about attrition bias and interpretability of the effect estimate. In contrast, Md Monoto et al. (2020), although also affected by substantial attrition, reported clear participant flow and outcome‐specific denominators at each follow‐up time point, allowing the data to be extracted and included in the meta‐analysis. Reeder et al. (2014) was excluded for specific outcomes because breastfeeding duration and exclusivity were derived from WIC recertification data, and outcome data were missing for participants who left WIC before their next scheduled recertification. In addition, the outcome denominators varied across time points, limiting direct pooling with studies reporting single end‐point estimates. RCTs = randomised controlled trials.

Data were extracted independently by two reviewers using a piloted data extraction form that captured study characteristics (e.g., setting, sample size, population), intervention details (e.g., peer support modality, frequency, delivery platform), outcome measures, and results. Where data were incomplete or unclear, corresponding authors were contacted for clarification. A PRISMA flow diagram (Figure 1) presents the study selection process.

### 2.3 Risk of Bias and Quality Assessment

Risk of bias was assessed using the Cochrane Risk of Bias Tool version 2 (RoB 2), evaluating domains of randomisation, deviations from intended interventions, missing data, outcome measurement, and selective reporting (Cochrane Methods Bias 2025; Higgins et al. 2019). Two reviewers conducted independent assessments, with discrepancies resolved through consensus or a third reviewer. Studies were rated as *low risk*, *some concerns*, or *high risk*. Reporting quality was further assessed using the CONSORT checklist (Cuschieri 2019).

### 2.4 Data Synthesis and Meta‐Analysis

A narrative synthesis was initially performed following Cochrane guidance (Higgins et al. 2019), organising findings by intervention type (e.g., one‐to‐one peer mentoring, group‐based digital communities), breastfeeding outcomes, and study context. A meta‐analysis was conducted when at least three studies reported comparable quantitative outcomes amenable to pooling. To ensure methodological transparency and data integrity, two studies meeting the inclusion criteria were excluded from the quantitative meta‐analysis. Meglio et al. (2010) was excluded because a high dropout rate in the intervention group and inconsistent intervention delivery raised concerns about attrition‐related bias and the interpretability of the effect estimate. Reeder et al. (2014) was excluded for specific outcomes because breastfeeding duration and exclusivity were derived from WIC recertification data, and outcome data were missing for participants who left WIC before their next scheduled recertification. In addition, the outcome denominators varied across time points, limiting direct pooling with studies reporting single end‐point estimates. Both studies were retained in the narrative synthesis to preserve contextual insights and ensure comprehensive interpretation of the evidence base.

Pooled risk ratios (RRs) with 95% confidence and prediction intervals were estimated using random‐effects models to account for between‐study heterogeneity (DerSimonian and Laird 1986) in R version 4.5.2 (R 2025), with statistical significance set at *p* < 0.05. Heterogeneity was quantified using the *I* ^2^ statistic, with values exceeding 50% indicating substantial heterogeneity (Higgins et al. 2003). Given the small number of contributing studies and the presence of imprecise estimates and dominant large comparisons in some analyses, *I* ^2^ values were interpreted cautiously as potentially underpowered indicators of true between‐study variability rather than definitive evidence of homogeneity. In the primary meta‐analysis, one effect size per study was used to avoid unit‐of‐analysis errors arising from repeated measures within the same participant cohort; time‐specific estimates from the same studies were presented descriptively rather than pooled across follow‐up points.

### 2.5 Sensitivity and Publication Bias Analyses

Sensitivity analyses were conducted based on the time frame for EBF. Publication bias was assessed using funnel plots (Egger et al. 1997).

## 3 Results

### 3.1 Study Selection and Characteristics

A total of seven studies, encompassing 3591 participants, met the inclusion criteria and were included in the quantitative synthesis, and five of these studies were eligible for quantitative meta‐analysis (*n* = 1565). The included studies were published between 2010 and 2022 and conducted across high‐ and upper‐middle–income countries, specifically Malaysia, Japan, the United States (three studies), Australia, and Hong Kong SAR, China. No studies were conducted in low‐income countries.

Across the included studies, interventions primarily delivered peer support via mobile applications, incorporating features such as group chat functions, peer discussion forums, real‐time messaging with peer supporters, and reminders aimed at reinforcing breastfeeding intentions and behaviours. Control groups typically received standard care, non‐peer mHealth education, or routine postpartum counselling. Key study characteristics are summarised in Table 2 and Supporting Information S1: Material 3.

**Table 2:** Characteristics and references of included studies.

Characteristic	*n* (%)
Study design:	
Randomised control trials (RCTs)^a^, ^b^, ^c^, ^d^, ^e^, ^f^, ^g^	7 (100.0%)
Pilot study^c^, ^g^	2 (28.6%)
Country:	
Australia^f^	1 (14.3%)
Hong Kong SAR, China^g^	1 (14.3%)
Japan^b^	1 (14.3%)
Malaysia^a^	1 (14.3%)
United States^c^, ^d^, ^e^	3 (42.9%)
HICs rate (Total)*	6 (85.7%)
UMICs rate (Total)*	1 (14.3%)
LMICs rate (Total)*	0 (0.0%)
Publication Year:	
2020‐2025	4 (57.1%)
< 2020 (i.e. 2019)	3 (42.9%)
Perinatal Period:	
Postpartum	4 (57.1%)
Perinatal Period	3 (42.9%)
Intervention categories:	
Telephone‐Based Support	5 (71.4%)
Mobile applications combined via live group support with educational contents	2 (28.6%)
Intervention contents:**	
Peer emotional/advice support via phone	5 (71.4%)
Structured group education sessions	1 (14.3%)
Digital group interaction	1 (14.3%)
Frequently asked questions and corresponding answers	1 (14.3%)
Multilingual support (Two languages or more)	1 (14.3%)
Strategies for working mothers	1 (14.3%)
Main outcomes:	
Exclusive Breastfeeding (EBF) rate*** (Total studies)	5 (71.4%)
− At 1‐2 months postpartum	3 (42.9%)
− At 3‐4 months postpartum	4 (57.1%)
− At 5‐6 months postpartum	4 (57.1%)
Any Breastfeeding rate (Total studies)	5 (71.4%)
− At 1‐2 months postpartum	3 (42.9%)
− At 3‐4 months postpartum	4 (57.1%)
− At 5‐6 months postpartum	4 (57.1%)
Other relevant outcomes:	
Breastfeeding self‐efficacy (2 months postpartum)	1 (14.3%)
Breastfeeding duration and frequency	3 (42.9%)
Breastfeeding initiation	1 (14.3%)
Breastfeeding attitude and satisfaction	2 (28.6%)

* LMICs: low‐and‐middle‐income countries; UMICs: Upper‐middle income countries; HICs: High‐income countries.

** Percentages do not sum to 100% as individual studies often incorporate multiple intervention components.

*** EBF rates are reported with specified period only, not including those without clearly mentioned period or mixed within any form of BF, i.e. mixed feeding.

a Md Monoto et al. 2020.

b Hongo et al. 2020.

c Cauble et al. 2021.

d Meglio et al. 2010.

e Reeder et al. 2014.

f Forster et al. 2019.

g Fan et al. 2022.

### 3.2 Narrative Synthesis of Studies

Telephone‐based and digital peer support interventions via mHealth show variable efficacy in sustaining breastfeeding among diverse mothers, with stronger effects on any breastfeeding duration than exclusivity in most cases. Studies in middle‐ to high‐income settings highlight benefits from personalised, timely peer guidance, though barriers like dropout and engagement persist. Narrative synthesis reveals contextual facilitators such as cultural congruence and peer relatability, underscoring the need for tailored delivery.

Peer support primarily used proactive phone calls post‐discharge (Md Monoto et al. 2020; Hongo et al. 2020; Cauble et al. 2021; Meglio et al. 2010; Reeder et al. 2014; Forster et al. 2019), with one asynchronous WhatsApp group (Fan et al. 2022). Interventions targeted high‐risk groups like adolescents or low‐income mothers (Meglio et al. 2010; Reeder et al. 2014) or primiparas (Cauble et al. 2021; Forster et al. 2019), often starting prenatally or early postpartum for real‐time troubleshooting on latching and supply.

Any breastfeeding rates improved significantly in several trials, such as 75% vs 69% (95% CI 1.02, 1.18) at 6 months (Forster et al. 2019) and nonexclusive ≥ 6 months among Spanish speakers (Reeder et al. 2014). EBF gains were substantial and subgroup‐specific, e.g. 73.3% vs 50.0% (*p* = 0.010) at 6 months overall (Md Monoto et al. 2020) and extended EBF duration for early adherers (Meglio et al. 2010), but null in others (Cauble et al. 2021; Fan et al. 2022). Lifestyle satisfaction rose notably (Hongo et al. 2020), yet overall EBF effects remained inconsistent across contexts.

High maternal satisfaction (76.7%–100%, *p* = 0.001, Md Monoto et al. 2020) and recommendation rates stemmed from relatable peers aiding work‐life balance (Hongo et al. 2020) and cultural/language fit (Reeder et al. 2014). Manuals and group empowerment boosted confidence in novices (Cauble et al. 2021), while early, sustained contacts reduced cessation hazards (Forster et al. 2019).

Dropouts reached 44.8% from uncontactability (Md Monoto et al. 2020), with connectivity and work conflicts noted (Cauble et al. 2021; Forster et al. 2019). Null effects were linked to high baselines, mixed‐feeding norms, or small samples (Hongo et al. 2020; Fan et al. 2022), alongside resource demands for peer training (Meglio et al. 2010).

### 3.3 Meta‐Analysis

#### 3.3.1 Exclusive Breastfeeding (EBF)

**Figure 2:**
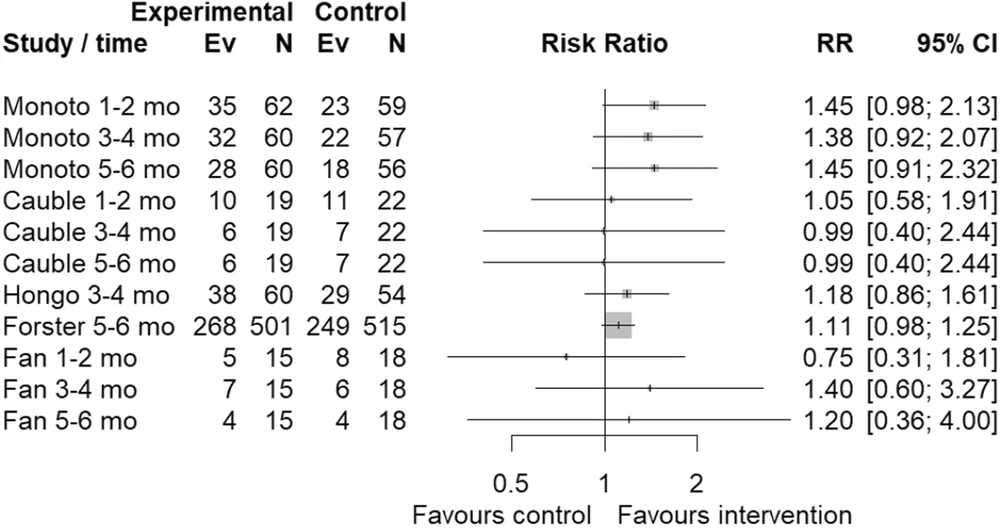
Forest plot‐ EBF by time. Note: Each row shows a time‑specific effect estimate (risk ratio, RR) and 95% confidence interval (CI) from an individual study at the indicated postpartum time point; the square markers indicate the point estimate and are scaled to study weight, and horizontal lines are 95% CIs. These rows are presented descriptively by time period (1–2, 3–4, and 5–6 months) and are not pooled together across different time points to avoid unit‑of‑analysis errors from repeated measurements in the same trial.

**Figure 3:**
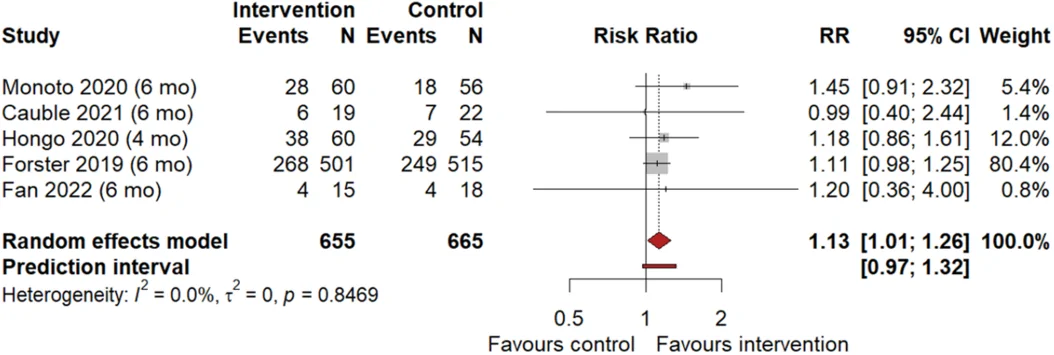
Forest plot‐ EBF (4–6 months postpartum). Note: This forest plot presents the pooled random‑effects meta‑analysis of exclusive breastfeeding (EBF) measured at 4–6 months postpartum using one effect size per study (the last available measurement within the 4–6 month window). Each row lists the study label (first author, year), the number of EBF events and sample size for the intervention and control groups, the study‑level risk ratio (RR) and 95% confidence interval (CI). The square markers show each study's point estimate and are scaled by study weight; horizontal lines show 95% CIs. The diamond at the bottom represents the pooled RR and its 95% CI from the random‑effects model; the value below the diamond also reports the prediction interval where calculated. The vertical line at RR = 1 indicates no difference (values > 1 favour the intervention). Heterogeneity statistics (I^2^, τ^2^, and p‑value) are displayed under the plot; interpret I^2^ cautiously when only a small number of studies contribute.

In the primary analysis for EBF at 4–6 months postpartum, the pooled risk ratio was 1.13 (95% CI 1.01–1.26), indicating a statistically significant improvement associated with the intervention (Figure 3). Heterogeneity in this model was 0.0%, although this should be interpreted cautiously given the small number of included studies. Figure 2 presents the time‐specific study estimates across follow‐up points descriptively and was not pooled across time points to avoid unit‐of‐analysis errors arising from repeated measurements within the same trials.

#### 3.3.2 Any Breastfeeding Rate

**Figure 4:**
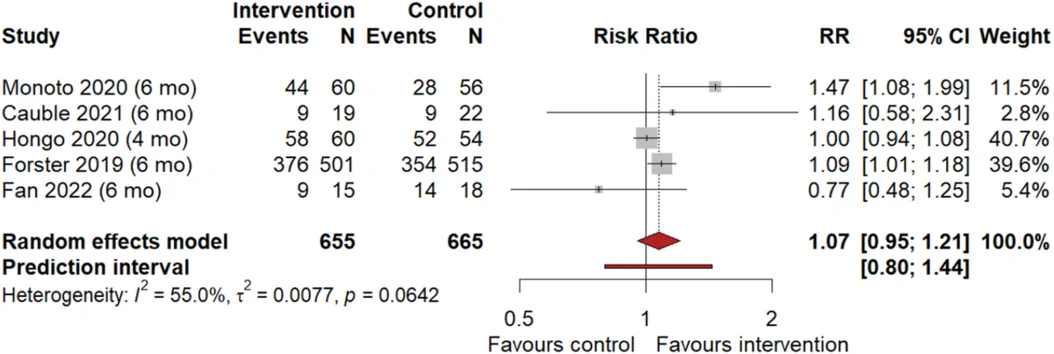
Forest plot‐ Any breastfeeding (4–6 months postpartum). Note: This forest plot shows the pooled random‑effects meta‑analysis of any breastfeeding at 4–6 months postpartum, using one effect size per study (last available measurement within the 4–6 month window). Each row reports the study label (first author, year), events and total (intervention vs control), the study‑level risk ratio (RR) and 95% confidence interval (CI). Square markers indicate point estimates (size proportional to study weight) and horizontal lines show 95% CIs; the diamond at the bottom represents the pooled RR and its 95% CI. The pooled estimate is shown with its prediction interval (in brackets) and heterogeneity statistics (I^2^, τ^2^, p‑value) beneath the plot; interpret heterogeneity cautiously given the small number of studies. The vertical line at RR = 1 denotes no difference; values > 1 favour the intervention.

**Figure 5:**
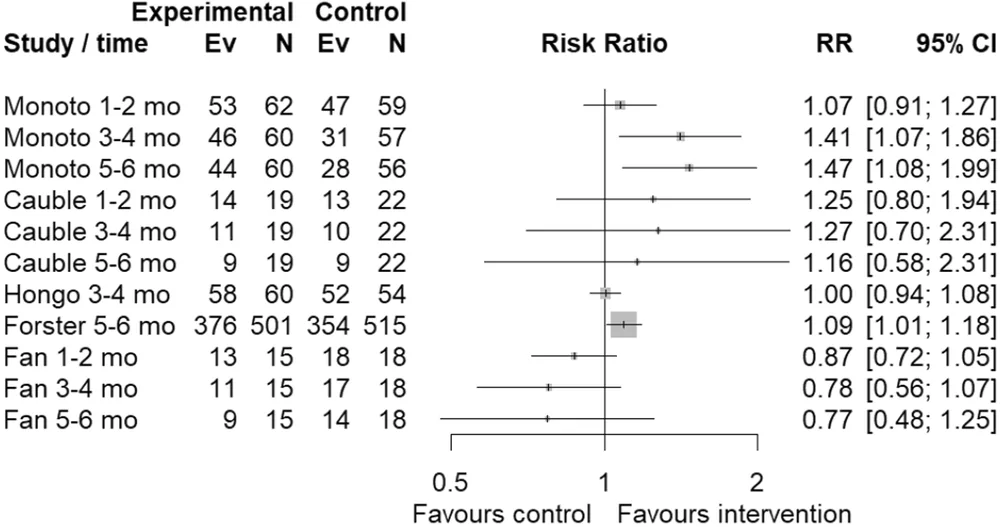
Forest plot‐ Any breastfeeding by time. Note: Each row shows a study‑level risk ratio (RR) and 95% confidence interval (CI) for any breastfeeding at the indicated time point (events/N for intervention and control groups are displayed). Square markers indicate point estimates (size proportional to study weight) and horizontal lines show 95% CIs. The vertical line at RR = 1 denotes no difference; values to the right favour the intervention.

In the primary analysis for any breastfeeding at 4–6 months postpartum, the pooled risk ratio was 1.07 (95% CI 0.95–1.21), indicating no statistically significant effect of the intervention at this time point (Figure 5). Between‐study heterogeneity was moderate (*I* ^2^ = 55.0%), and the prediction interval crossed the null, suggesting that effects may vary across studies and settings. Figure 4 presents the time‐specific study estimates across follow‐up points descriptively and was not pooled across time points to avoid unit‐of‐analysis errors arising from repeated measurements within the same trials.

### 3.4 Publication Bias and Leave‐One‐Out Analyses

The funnel plots for any breastfeeding and EBF were presented as exploratory assessments of potential small‐study effects or publication bias (Supporting Information S1: Material 4). The leave‐one‐out analyses (Supporting Information S1: Material 5) showed that the summary effect of pooled estimate for any breastfeeding remained close to the null and heterogeneity persisted at a moderate level. For EBF, the leave‐one‐out results were similarly robust, with the pooled effect remaining statistically significant across all iterations and heterogeneity remaining absent (*I* ^2^ = 0%). However, this finding should be interpreted in light of the small number of included studies, which may have limited the power to detect true between‐study differences.

### 3.5 Risk of Bias and Quality Assessment

The risk of bias was assessed using the Cochrane Risk of Bias 2.0 (RoB 2) tool (Cochrane Methods Bias 2025) across five domains, as shown in Table 3. Although most studies were judged as low risk overall, concerns related to missing outcome data and attrition were noted in some trials, particularly Meglio et al. (2010), which reported substantial dropout. Overall risk‐of‐bias judgments should therefore be interpreted with caution and in conjunction with study retention patterns. All RCTs adhered to CONSORT reporting standards (Cuschieri 2019).

**Table 3:** Risk of bias and quality assessment.

		Md Monoto et al. (2020)	Hongo et al. (2020)	Cauble et al. (2021)	Meglio et al. (2010)	Reeder et al. (2014)	Forster et al. (2019)	Fan et al. (2022)
● Green = Low risk	Q1	●	●	●	●	●	●	●
● Yellow = Some concerns	Q2	●	●	●	●	●	●	●
	Q3	●	●	●	●	●	●	●
	Q4	●	●	●	●	●	●	●
	Q5	●	●	●	●	●	●	●
	Overall risk of bias	●	●	●	●	●	●	●
	CONSORT adherence	●	●	●	●	●	●	●

**Q1:** Bias arising from the randomisation process (sequence generation, concealment, baseline differences).

**Q2:** Bias due to deviations from intended interventions (blinding, adherence to assigned intervention).

**Q3:** Bias due to missing outcome data (completeness of data, reasons for missing data).

**Q4:** Bias in measurement of the outcome (blinding of assessors, validity of measurement tools).

**Q5:** Bias in selection of the reported result (selective reporting, analysis per pre‐specified plan)

*Note:* Risk of bias was assessed independently by two reviewers using RoB 2 across five domains, with overall judgements based on the highest level of concern in any domain. Reporting quality was also reviewed using the CONSORT checklist. Of the 7 studies included, 4 (57.1%) were judged to have low overall risk of bias, while 3 (42.9%) had some concerns. Cauble et al. (2021) and Reeder et al. (2014) had some concerns in the domain of missing outcome data, and Meglio et al. (2010) had some concerns in the domains of deviations from intended interventions and missing outcome data. All RCTs adhered to CONSORT reporting standards (Cuschieri 2019). Meglio et al. (2010) and Reeder et al. (2014) were retained in the narrative synthesis and included in this table, although they were excluded from the quantitative meta‐analysis.

## 4 Discussion

This systematic review and meta‐analysis provides an updated synthesis suggesting that peer support delivered through mobile health applications may improve EBF, particularly at 4–6 months postpartum, although no statistically significant pooled effect was observed for any breastfeeding at the primary time point. The findings suggest that digital peer support may be especially effective for sustaining more demanding breastfeeding behaviours, while its effect on broader breastfeeding continuation is less consistent across studies.

The observed benefit for EBF is consistent with prior evidence that breastfeeding success depends not only on information, but also on emotional encouragement, practical advice, and reassurance from women with lived breastfeeding experience. (McFadden et al. 2017; Trickey 2018). Unlike traditional peer support programmes that rely on in‐person interactions, mHealth‐based peer support provides scalable, real‐time, and contextually adaptable engagement opportunities through mobile applications integrating social networking elements, message boards, and group mentoring systems that promote collective learning, social connectedness, maternal confidence, normalisation of breastfeeding challenges, and sustained motivation via continuous feedback and shared experiences, which are associated with improved breastfeeding duration and self‐efficacy (Shakya et al. 2017).

By contrast, the pooled result for any breastfeeding was not statistically significant at the primary time point, despite several individual trials showing favourable effects. This discrepancy likely reflects clinical and methodological differences across studies, including intervention intensity, timing of outcome assessment, participant characteristics, and platform type. It also suggests that maintaining any breastfeeding may require more sustained or comprehensive support than the interventions in the included trials were able to provide, especially when mothers face work‐related pressures, mixed‐feeding norms, or early return to usual routines. The low I^2^ values (0%) should be interpreted cautiously, as they likely reflect limited statistical power to detect heterogeneity given the small number of studies and comparisons, several imprecise estimates with wide confidence intervals, dominance of one large study (Forster et al. 2019 carrying 80.4% weight in EBF 4–6 months), and small‐sample bias in *I* ^2^ estimation. Moderate heterogeneity observed for any breastfeeding further supports using random‐effects models as the primary approach. These results extend previous reviews focusing on general mHealth interventions (DeNicola et al. 2020; Qian et al. 2021; Ziebart et al. 2024), providing more targeted evidence on the unique value of peer‐to‐peer digital engagement as a behavioural mechanism for improving breastfeeding continuity.

### 4.1 Comparison With Previous Literature

Compared with previous literature, the present review provides more targeted evidence that peer support delivered through mobile health applications is beneficial for breastfeeding, particularly EBF. Telephone‐based support dominated (71.4% of studies), providing proactive emotional and advisory aid via scheduled calls, while digital formats like WhatsApp groups offered interactive Q&A and experience‐sharing. These interventions boosted EBF across postpartum periods (4–6 months) and secondary outcomes like self‐efficacy and satisfaction, aligning with prior evidence from McFadden et al. (2017) and Shakya et al. (2017) on peer support's role in sustaining breastfeeding in resource‐limited contexts.

Earlier reviews of broader mHealth interventions reported inconsistent or modest effects, suggesting that digital breastfeeding support is not uniformly effective across outcomes or contexts (Chen et al. 2018; Sondaal et al. 2016; Daly et al. 2018). Our findings indicate that the peer‐support element may be an important differentiator, because it combines practical advice with emotional reassurance and social connectedness, which are known to support maternal breastfeeding confidence. The present results also align with earlier peer‐support research showing that interpersonal encouragement and shared lived experience can improve breastfeeding continuation (Gavine et al. 2022; Jolly et al. 2012). However, our pooled analysis found no significant overall effect on any breastfeeding at the primary time point, despite several favourable individual trial results. This divergence may reflect differences in intervention format, outcome measurement, follow‐up timing, and participant characteristics, as well as barriers such as dropout, digital inequity, and work‐related constraints.

### 4.2 Generalisability Beyond High‐ and Upper‐Middle‐Income Settings

Consistent with the scoping review by Chepkirui et al. (2020), which noted that most evidence on breastfeeding peer support originates from high‑income countries, our meta‑analysis is similarly dominated by trials from high‑ and upper‑middle‑income settings, limiting generalisability to low‑resource contexts where smartphone accessibility, data costs, connectivity, and cultural perceptions of online support may differ substantially. Previous non‑digital peer support research in low‑ and middle‑income countries has shown variable results, often constrained by programme sustainability and limited health system infrastructure (McFadden et al. 2017), underscoring that even well‑designed support models can perform differently when implemented in more fragile contexts. Within our included studies, the predominance of high‑income samples (85.7%) and features such as multilingual options (14.3%) and work–life strategies (14.3%) highlights the potential scalability of mHealth for overcoming traditional peer‑support barriers, such as geographic access and time demands, particularly for primiparas and working mothers, and peer relatability appeared to foster confidence in these higher‑resource groups. At the same time, emerging tele‑intervention and mHealth studies among low‑income women show that socio‑structural barriers, including affordability, competing survival priorities, and fragmented service integration, can substantially shape both feasibility and impact of remote breastfeeding support. Consequently, the promising but preliminary evidence summarised here should not be assumed to apply globally; rather, it points to the need for context‑specific adaptation and rigorous evaluation of mHealth peer support in low‑resource settings, co‑designed with local mothers and health systems and explicitly oriented towards closing, rather than widening existing digital and social equity gaps.

### 4.3 Digital Divide and Equity Considerations

Although mHealth platforms are often framed as scalable and low‐cost channels for peer support, mobile peer‑support interventions are not automatically equitable for all mothers in high‑income settings (Al Dahdah 2017; Pierce et al. 2023). For women relying on prepaid data plans or unstable home internet, regular use of group messaging apps or mobile platforms can quickly become unaffordable or technically unreliable, making sustained participation difficult (Al Dahdah 2017; Jonayed and Rumi 2024). Qualitative studies on digital maternal health also describe how limited private device access, competing caregiving demands, and worries about confidentiality can reduce willingness to engage with online breastfeeding support, particularly among more vulnerable groups (Pierce et al. 2023; Udenigwe and Yaya 2022; Udenigwe et al. 2023). In practice, these conditions mean that the mothers who are most digitally and socioeconomically advantaged are often best placed to benefit from mHealth peer support, while those facing the greatest barriers to breastfeeding are least able to use it consistently. Addressing affordability, low‑data or offline functionality, and inclusive design is therefore essential if mobile peer interventions are to contribute to, rather than undermine, breastfeeding equity (Al Dahdah 2017; Jonayed and Rumi 2024; Udenigwe et al. 2023).

### 4.4 Heterogeneity of Intervention Components

Across the included trials, there was substantial variation in intervention design, including mode of delivery (proactive one‑to‑one telephone calls, app‑mediated messaging, and moderated group chats), intensity and duration of contact, and the level and type of training provided to peer supporters (Supporting Information S1: Material 3). However, the small number of studies, diverse operationalisations of “peer support,” and incomplete reporting of implementation details mean that the current evidence base is too sparse to determine which specific components, such as proactive versus reactive contact, lay versus professional support, or group versus individual delivery, are most effective for improving breastfeeding outcomes. Future trials should therefore pre‐specify and rigorously report intervention mechanisms, enabling more informative cross‐study comparisons and meta‐regression approaches to disentangle which combinations of features work best for whom.

### 4.5 Engagement, Attrition, and Real‐World Scalability

Engagement with mHealth interventions was variable, with some trials reporting high dropout rates. For example, up to 44.8% loss to follow‐up in one telephone peer‑counsellor programme, raising concerns about internal validity and scalability. This pattern is consistent with broader mHealth literature, in which attrition rates of 17%–26% are commonly reported and tend to be higher for app‐based interventions than for simpler message‐based approaches. Nonusage attrition is often concentrated among adolescents and socioeconomically disadvantaged users, who may disengage early because apps are perceived as unhelpful, burdensome, or misaligned with their daily realities. In the context of breastfeeding peer support, high dropout, difficulties sustaining participation over months, and competing maternal demands suggest that digital peer interventions must be paired with robust engagement strategies, such as blended human–digital models, tailored prompting, and equity‐informed design to avoid overestimating effectiveness in tightly controlled trials relative to real‐world implementation.

### 4.6 Strengths and Limitations

This review demonstrates several strengths, including the exclusive inclusion of randomised controlled trials, rigorous PRISMA‐compliant methods, robust sensitivity analyses, and generally low risk of bias across studies (85.7%), with strong PRISMA adherence.

However, important limitations should be acknowledged. First, the small number of trials (k = 7) restricts statistical power for exploring key moderators such as maternal age, parity, or prior breastfeeding experience. Mean or median maternal ages varied across studies, and some trials did not report age clearly, further limiting the precision of age‑specific interpretations. Second, although peer support delivery varied meaningfully across interventions, key implementation features—including peer training content, interaction frequency, contact duration, and app functionality—were not consistently reported across studies. These components were not consistently or comprehensively reported, constraining inferences about which features are most important for effectiveness. Third, the evidence base is narrow in scope: the review was limited to English‑language publications, no low‑income country trials were identified (0%), and two eligible studies were retained in the narrative synthesis but excluded from the quantitative meta‐analysis because of methodological limitations, including substantial attrition and non‐uniform outcome denominators across follow‐up time points. These factors reduce generalisability and may introduce language, retention, and comparability bias.

Measurement issues also warrant caution. Primary breastfeeding outcomes in the included trials relied largely on maternal self‑report, making them vulnerable to recall bias and social desirability bias. Secondary outcomes, such as breastfeeding self‑efficacy, attitudes, and knowledge, were operationalised with different instruments and definitions across studies, preventing a robust, standardised synthesis. Moreover, follow‑up rarely extended beyond 6 months, so there is limited evidence on whether digital peer networks can sustain breastfeeding behaviours over longer periods, or influence continued partial breastfeeding beyond the early postpartum window. From a statistical perspective, the observed *I* ^2^ = 0% for EBF should be interpreted cautiously, as it may reflect limited power to detect between‐study heterogeneity rather than true homogeneity, particularly given the small number of included studies and the dominance of one large comparison.

Finally, participant characteristics related to socioeconomic status and digital access were incompletely reported in several trials. Important indicators, such as household income, employment conditions, housing stability, mobile data plan type, and whether devices were personally owned or shared, were often missing or only crudely described, and very few studies stratified outcomes by socioeconomic position or digital access. As a result, it is unclear whether the observed effects chiefly reflect relatively advantaged, well‑connected mothers or extend to those facing compounded social and digital disadvantage.

## 5 Recommendations and Conclusion

This systematic review and meta‐analysis suggests that peer support delivered through mobile health applications can improve EBF among postpartum mothers, particularly at 4–6 months postpartum, but its effect on any breastfeeding is less consistent. The evidence indicates that mHealth‐based peer support is a promising approach for strengthening breastfeeding confidence, satisfaction, and perceived support, especially when interventions are interactive, timely, and culturally relatable.

Despite these benefits, evidence remains geographically concentrated in HICs and technologically focused, limiting global generalisability due to potential barriers like digital inequities, high dropouts and reliance on self‐reports; this highlights needs for cultural relevance, infrastructure evaluation, and addressing engagement challenges in diverse populations. Practice and policy should consider integrating structured digital peer support into maternal mHealth programmes, with quality standards for peer‐training protocols and culturally appropriate content to boost self‐efficacy. Future large‐scale, multicentre RCTs should prioritise LMICs, hybrid designs with real‐time video, long‐term EBF tracking, process evaluations of engagement or trust dynamics, and cost‐effectiveness analyses to inform WHO guideline integration and national breastfeeding strategies. More granular socioeconomic and digital access data should be collected, and explicitly examine how pay‑as‑you‑go plans, limited data, unstable connectivity, and lower digital literacy influence recruitment, retention, and the effectiveness of mHealth peer support interventions, including in settings where digital divides are most pronounced, and offer a promising, scalable complement to traditional services, advancing global maternal–infant health goals.

## Author Contributions

C.Y. conceived and designed the study, developed the methodology, and supervised the research team with consultation with K.S. and M.F. A review team consisting of C.Y., M.W., P.L. and K.S. performed the screening and data extraction, C.Y. analysed the data. C.Y. drafted the initial manuscript and incorporated critical revisions suggested by all co‐authors. All authors reviewed and approved the final version of the manuscript and agreed to be accountable for all aspects of the work to ensure its accuracy and integrity.

## Conflicts of Interest

The authors declare no conflicts of interest.

## Supporting information


Supporting File 1

Supporting File 2

Supporting File 3

Supporting File 4

Supporting File 5

## Data Availability

The data that support the findings of this study are available from the corresponding author upon reasonable request.
