# Supplementary material for: The Impact of Peer Support Through Mobile Health Applications on Breastfeeding Outcomes: A Systematic Review and Meta‐Analysis
Source: Matern Child Nutr. 2026 Sep 21;22(3):e70249. doi: 10.1111/mcn.70249 (PMC13591216; doi:10.1111/mcn.70249)
Supplement: Supplementary file 1 [file MCN-22-e70249-s001.docx]

Supplementary Material 4. Funnels plots for Any breastfeeding and EBF.

Note: Each funnel plot displays study effect sizes (log risk ratio) on the x‑axis against their standard error (y‑axis); individual studies are plotted as points and the vertical line represents the pooled effect estimate. These plots are presented as exploratory visual checks only: with few studies (k≤5) funnel‑plot interpretation is underpowered and asymmetry may reflect heterogeneity or chance rather than publication bias.
