# Supplementary material for: The Impact of Peer Support Through Mobile Health Applications on Breastfeeding Outcomes: A Systematic Review and Meta‐Analysis
Source: Matern Child Nutr. 2026 Sep 21;22(3):e70249. doi: 10.1111/mcn.70249 (PMC13591216; doi:10.1111/mcn.70249)
Supplement: Supplementary file 2 [file MCN-22-e70249-s002.docx]

Supplementary material 2. PICO framework.

| Population | Intervention | Comparator | Outcome |
| --- | --- | --- | --- |
| Pregnant women and postpartum (up to one year) mothers | mHealth interventions delivered via mobile applications, smartphones, text messaging (SMS), telemedicine, eHealth, web-based, or other digital platforms | Standard care, usual practice, or alternative non-mHealth interventions | Breastfeeding initiation, exclusivity, duration, self-efficacy, knowledge, satisfaction, and related behavioural changes or practices |
