# Supplementary material for: The Impact of Peer Support Through Mobile Health Applications on Breastfeeding Outcomes: A Systematic Review and Meta‐Analysis
Source: Matern Child Nutr. 2026 Sep 21;22(3):e70249. doi: 10.1111/mcn.70249 (PMC13591216; doi:10.1111/mcn.70249)
Supplement: Supplementary file 3 [file MCN-22-e70249-s003.docx]

Supplementary Material 5a. Leave-one-out analyses for Any breastfeeding.

Note: This sensitivity analysis shows the pooled random‑effects risk ratio (RR) for any breastfeeding after omitting one study at a time. Each row displays the summary effect, 95% confidence interval (CI), and heterogeneity estimates after exclusion of the named study. The diamond at the bottom represents the overall pooled estimate from the leave‑one‑out model; values to the right of RR = 1 favour the intervention. This analysis assesses whether the main finding is unduly influenced by any single study.

Supplementary Material 5b. Leave-one-out analyses for EBF.

Note: This sensitivity analysis shows the pooled random‑effects risk ratio (RR) for any breastfeeding after omitting one study at a time. Each row displays the summary effect, 95% confidence interval (CI), and heterogeneity estimates after exclusion of the named study. The diamond at the bottom represents the overall pooled estimate from the leave‑one‑out model; values to the right of RR = 1 favour the intervention. This analysis assesses whether the main finding is unduly influenced by any single study.
