# Supplementary material for: The Impact of Peer Support Through Mobile Health Applications on Breastfeeding Outcomes: A Systematic Review and Meta‐Analysis
Source: Matern Child Nutr. 2026 Sep 21;22(3):e70249. doi: 10.1111/mcn.70249 (PMC13591216; doi:10.1111/mcn.70249)
Supplement: Supplementary file 4 [file MCN-22-e70249-s004.docx]

**Supplementary material 3. Details of included studies (k=7).**

Note: n=number of participants; k=number of studies. The characteristics are limited to information explicitly reported in the included study reports available for extraction, where details that are not visible in the literature, cells are marked NR (not reported). For Abbreviations: ABA, Australian Breastfeeding Association; GBPC, group-based phone counseling; IBCLC, International Board Certified Lactation Consultant; MBfPC, Malaysian Breastfeeding Peer Counselor; MBfPCA, Malaysian Breastfeeding Peer Counselor Association; NICU, neonatal intensive care unit; RCT, Randomized control trials; WIC, Special Supplemental Nutrition Program for Women, Infants, and Children.

| Author/Year | Country/ setting | Study design and sample | Maternal/  sample characteristics reported | Mode/  platform | Peer characteristics / training | Comparator / usual care | Timing and duration | Peer support format and intensity | Attrition (dropout / loss to follow-up)  (%), overall | Notes relevant to diversity / representativeness |
| --- | --- | --- | --- | --- | --- | --- | --- | --- | --- | --- |
| Di Meglio et al., 2010 | United States (two hospitals in Rochester, New York) | RCT; n=78 adolescent mothers (<20 years) with singleton infants and uncomplicated postpartum courses | Adolescent mothers (<20 years), singleton births, uncomplicated postpartum course | Telephone peer support | Adolescent mothers with (>4 weeks) breastfeeding experience; 20-hour La Leche League-based training | Control participants received no telephone peer support, but both groups could access family/friends, pediatric providers, and hospital lactation consultants | Postdischarge calls at 2, 4, and 7 days, then at 2, 3, 4, and 5 weeks postdischarge | 1-to-1 telephone support; provided personal phone numbers and resource referrals | 41 | Population was highly specific to breastfeeding adolescents, which limits generalizability to older postpartum populations. Ethnicity information was only partially reported in the extracted text |
| Reeder et al., 2014 | United States (four Oregon WIC local agencies; urban and rural) | Stratified RCT; n=1948 prenatal WIC participants | Low-income prenatal WIC clients intending to breastfeed or undecided; stratified by English/Spanish language | Telephone peer counseling | Current/recent WIC clients with ≥ 6 months breastfeeding experience; trained via Loving Support curriculum | Standard WIC breastfeeding promotion/support without peer counselor contact | Began in pregnancy and continued postpartum. Low-frequency arm: 4 planned contacts (after prenatal assignment, 2 weeks before due date, 1 and 2 weeks postpartum). High-frequency arm: 8 planned contacts (same first 4 plus months 1, 2, 3, and 4 postpartum) | 1-to-1 proactive telephone support. Low-frequency (4 planned calls); High-frequency (8 planned calls) | 18.5 | Sample represented a socioeconomically disadvantaged public-health population, but detailed baseline characteristics were not fully available in the extracted text. Language/acculturation was an important subgroup factor. |
| Forster et al., 2019 | Australia (three public hospitals in Victoria) | Multicentre RCT; n=1152 women | Primiparous women from relatively disadvantaged public hospitals intending to breastfeed; English proficient | Proactive telephone-based peer support | Peer volunteers with ≥ 6 months breastfeeding experience (<8 hours professional training); 4-hour adapted ABA training with monthly supervision | Usual postpartum hospital and community breastfeeding support, including lactation consultants, postnatal home visits, maternal-child health nurse service, and access to ABA helpline | First call 24–48 hours after discharge, second call 3–4 days later, weekly calls for first 12 weeks, then every 3–4 weeks until 6 months postpartum | 1-to-1 proactive telephone support for up to 6 months (weekly for first 12 weeks, then every 3–4 weeks | 11.8 | Included primiparous women from public hospitals serving relatively disadvantaged women, but participants were English-proficient and breastfeeding intention was high, which may limit transferability to more diverse or less motivated populations. |
| Hongo et al., 2020 | Japan (four maternity hospitals in Tokyo and Kanagawa metropolitan areas) | Randomized longitudinal trial; n=114 mothers | Urban sample with high education (>83%), low smoking rates, 100% partner cohabitation, ~50% multiparity, and 52–56% prenatal exclusive breastfeeding intention | Telephone-based peer support | The extracted text identifies the intervention as telephone-based peer support, but detailed peer eligibility and training characteristics were not available in the extracted text | Conventional support at participating hospitals | Support from hospital discharge until 4 months postpartum; outcomes assessed at 1 and 4 months postpartum | NR | 8.8 | Urban Japanese sample with universal insurance context and very high partner cohabitation. Socioeconomic detail was limited to education and financial worry. |
| Md Monoto et al., 2020 | Malaysia (national tertiary teaching hospital; UKMMC) | Single-blinded parallel RCT; n=210 postpartum mothers | Healthy postpartum mothers with term, singleton vaginal births; proficient in Malay and/or English | Telephone-based peer counseling | Secondary school graduates with ≥6 months personal breastfeeding experience; 3-day structured MBfPC training | Routine care (midwife advice, pamphlets, MBfPCA info) with independent access to providers, lactation consultants | Contact within 72 hours after discharge, then at 2 weeks, and at 1, 2, 4, and 6 months postpartum; extra calls allowed if needed | One-to-one telephone support with minimum scheduled contacts plus additional participant-initiated or peer-judged support. Interaction logs were maintained | 44.8 | Sample was hospital-based and selectively healthier postpartum mothers; loss to follow-up was substantial. Limited extracted reporting constrains description of ethnicity and socioeconomic diversity |
| Cauble et al., 2021 | United States (Kansas City metropolitan obstetrics/gynecology practice) | Pilot randomized controlled feasibility trial; n=45 women | Primiparous or prior EBF <3 months, aged 18–35. Demographics collected but distributions NR. Excluded high-risk pregnancies, multiple gestations, and chronic metabolic/gastrointestinal conditions | Group-based phone counseling via audio conference system | Sessions were led by an International Board Certified Lactation Consultant and registered dietitian, not by peer mothers | Usual pregnancy and pediatric education from healthcare providers; no additional breastfeeding/nutrition education | Six weekly sessions delivered prenatally between 16 and 30 weeks’ gestation; no postpartum peer support component | Group-based phone counseling, approximately 60 minutes/session, with 6–10 participants per group. Lessons covered breastfeeding basics, pumping, return to work, solids, and maternal nutrition/physical activity | 8.9 | Small, clinic-based pilot sample with restrictive eligibility criteria; likely not representative of higher-risk or medically complex pregnancies |
| Fan et al., 2022 | Hong Kong (antenatal clinic in a public hospital) | Single-blinded, open-label pilot RCT; n=33 primiparous women | Primiparous Cantonese-speaking Hong Kong residents with healthy, singleton term pregnancies | WhatsApp online peer-support group | Three trained peer counselors hosted the group. Peer supporters were women with at least 2-month breastfeeding experience and were trained under the Department of Health in Hong Kong to provide breastfeeding peer support | Standard care without the WhatsApp peer-support group | WhatsApp group addition ≤ days; weekly peer prompts for 6 months. Multi-point phone follow-up (months 1, 2, 4, 6) for both arms | Group-based interactive WhatsApp platform; weekly peer-led prompts and participant interaction for 6 months | 3.0 | Sample was small, linguistically specific, and limited to healthy Cantonese-speaking Hong Kong residents, reducing broader representativeness |
